# The Evaluation of the First Bahname Written in Turkish in the Ottoman Era Concerning Current Urology

**DOI:** 10.5152/tud.2022.22104

**Published:** 2022-11-01

**Authors:** Muhammet Ihsan Karaman, Adem Az

**Affiliations:** 1 Department of Medical History and Ethics, İstanbul Health and Technology University, İstanbul, Turkey; 2 Department of Medical History and Ethics, İstanbul University Faculty of Medicine, İstanbul, Turkey

**Keywords:** Bahname, sexual life, sexual health, urology, sexology, andrology

## Abstract

**Objective:**

Our study investigated the oldest known Turkish bahname, translated by Musa b. Mes’ud, in comparison with the current literature.

**Material and methods:**

First, the original manuscript of the translation was transcribed in Latin. The final version of the text was analyzed in the results. In discussion, findings were examined and interpreted within the framework of current knowledge of sexology, urology, and andrology.

**Results:**

Although the work mostly mentions supportive and therapeutic practices in sexual health, it also provides advice on sexuality and sexual life, discussing several topics regarding sexual intercourse types, explaining which ones are good or harmful, and their timing or frequency. The author recommends many foods and compounds or specific drugs and ointments to enhance sexual stamina and avoid erectile dysfunction. In addition, he also tries to find solutions to some other sexual health problems related to men and women. These issues are generally evaluated in the context of health; a religious perspective is also provided when needed.

**Conclusion:**

Interestingly, the author’s recommendations on sexual health and herbal or animal drugs are consistent with the current literature. Nevertheless, some information and suggestions in works are entirely irrational and unscientific. Consequently, this study is an original investigation of the first translated bahname into Turkish. There is no other study examining the bahnames with this method. Thus, we believe that our work will be a significant contribution to the research literature.

**Main Points**

- This is the first study that investigated the bahnames in comparison with the current literature.
- The author recommends many foods and compounds, or specific drugs and ointments, consistent with the current literature, to enhance sexual stamina and prevent erectile dysfunction.
- The author synthesized empirical information with experiences from the past in the bahname while dealing with sexual life and sexual health issues. These issues are generally evaluated in the context of health; a religious perspective is also provided when needed.

## Introduction

In the Islamic medical literature, works dealing with sexual health issues are called “bahname.” The word bahname is a combination of the Arabic word “bah,” which means “sexual desire, lust, libido,” and the Persian word “name,” meaning “book.”^1^ The Turkish Encyclopedia of Islam (TEI) defines bahname as “a type of book containing information about all kinds of sexual issues, including especially the treatment of sexual disorders.”

The bahnames, an essential part of the Islamic medical corpus, generally address sexuality, sexual behavior, sexual dysfunctions, and protective, supportive, and therapeutic applications in this field. Additionally, such books may include social issues such as sexual life, family institution, marriage and pregnancy, measures to facilitate or prevent conception, problems during pregnancy, medical information about newborn children, and even child-rearing and upbringing.^1^

Many physicians wrote such works in different periods of Islamic and Ottoman medical history. Ali Haydar Bayat identified 45 different bahnames across all periods of Islamic civilization, including 21 in Arabic, 6 in Persian, and 14 in Turkish.^2^ Ilter Uzel also listed 51 works in Arabic, Persian, and Turkish.^3,4^ Jabir ibn Hayyan, Jabril ibn Bukhtishu, al-Kindi, Hunayn ibn Ishaq, Qusta ibn Luqa, al-Razi, Ibn Sina, Ibn Maymun, Ibn al-Bitriq, and Shayzari were among the most prominent Islamic scholars who wrote bahnames. According to the TEI, the oldest bahname in Turkish is a translation of the *Bahname-i Padişahi* with a dedication to the Sarukhanid Ya‘qub b. Dawla in the fourteenth century. Additionally, the first known Turkish bahname in the Ottoman Empire is the translation by Musa b. Mes’ud with the same title as the original Persian version of Bahname-i Padisahi.^4^

Among the Bahnames in Ottoman era, especially those presented to the Sultanes are decorated with colorful miniatures, while those written for the public include no pictures or figures. The *Bahname-i Padişahi* translated by Murat b. Mesud is one of the first examples of the Ottoman era. Unlike most of the bahnames, it does not contain pictures and figures. One of the most famous samples which include figures was Cemaleddin Revnaki’s book called *Kitâbü’s-Safâ ve’s-surûr*. Bahnames included more figures and became more pornographic after the nineteenth century.^1,5^

This study investigated the first translated bahname in the Ottoman Empire and concepts such as sexuality, sexual life, sexual desire/function disorders, and the protective, supportive, and therapeutic applications in this work compared to the current literature.

## Materials and Methods

In this study, we submit the transcription of an original text in the Ottoman medical history and its comparative evaluation with the current literature. Therefore, we did not require any Ethics Committee Approval, and such a study is not within the scope of Clinical Research Ethics Committees.

**Figure 1.**
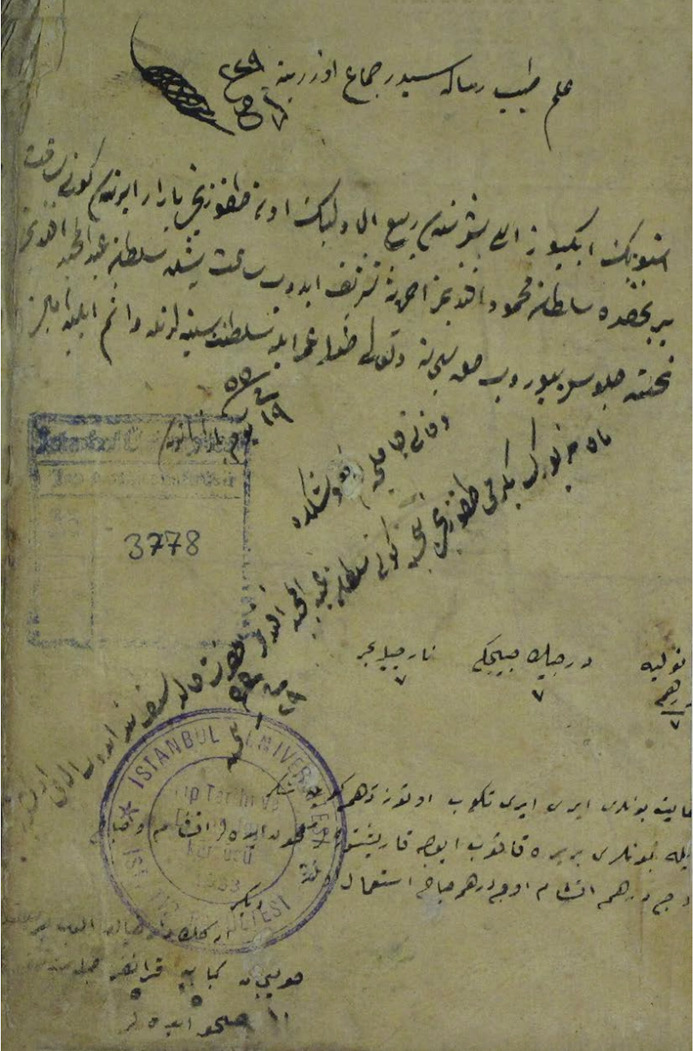
Cover image of the copy registered at the Library of Istanbul University Istanbul Faculty of Medicine under call number 3778.

**Figure 2.**
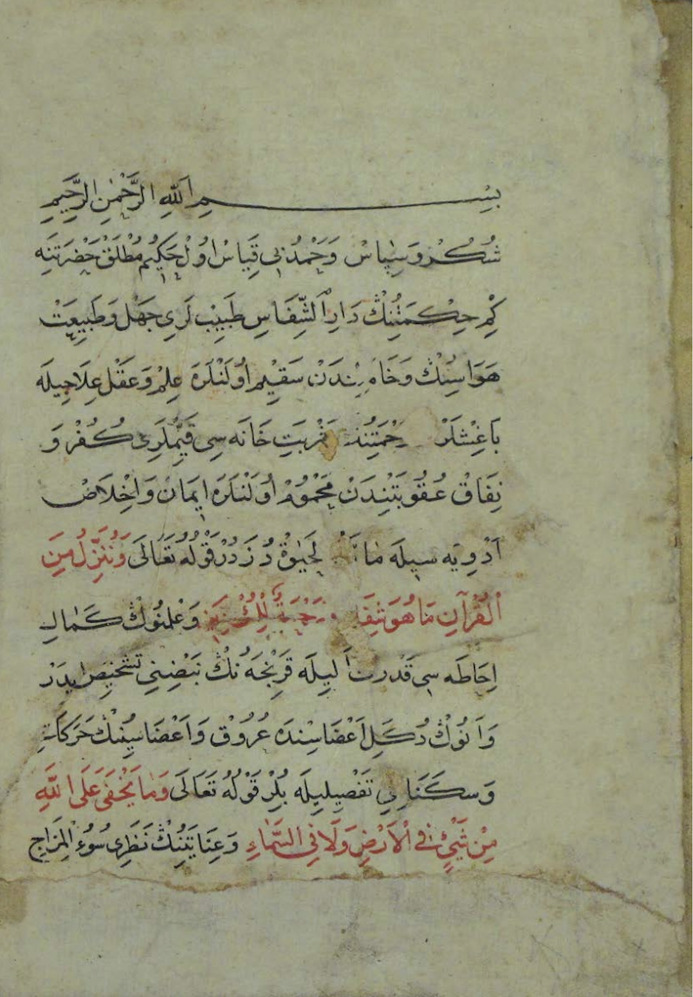
Example page from the manuscript that is investigated in our study.

The chapter and folio numbers mentioned in our study for the bahname that was translated by Musa b. Mes‘ud refers to the copy registered in the Library of Istanbul University Istanbul Faculty of Medicine under call number 3778, 1 of 5 known manuscripts of this manuscript (Figures 1 and 2).

First, the original manuscript of the translation was transcribed in Latin. Parts that could not be read due to the damages were read from the copies of the Suleymaniye Library, Sehid Ali Pasha Library, and the National Library and added to the transcription text. The final version of the transcribed text was analyzed in the results. In discussion, findings were examined and interpreted within the framework of current knowledge of sexology, urology, and andrology.

## Results

According to the index quoted by Uzel with reference to Sehsuvaroglu, Musa b. Mes‘ud’s bahname translation consists of 17 chapters.^3,6^ Table 1 shows the index of the bahname.

The topics of bahname can be grouped into 2 categories:

1. Issues related to sexuality and sexual life
2. Protective, supportive, and therapeutic applications for the protection of sexual life.

### Issues Related to Sexuality and Sexual Life

Although the work mainly mentions supportive and therapeutic practices related to sexual health, it also provides advice on sexuality and sexual life, discussing several topics regarding sexual intercourse types, explaining which ones are good or harmful, and their timing or frequency. These issues are generally evaluated in the context of health, and a religious perspective is also provided when needed.

Folio-5a emphasizes that spouses are religiously permitted to have sexual intercourse in whatever they want. It also defined and recommended many different sexual intercourse types such as standing, sitting, and being on top or at the bottom. In addition, the work expresses explicit prohibitions against homosexuality and anal sex.

In the fourteenth chapter, first, the author describes the beneficial and beautiful sexual intercourse types, then defines the following as the types of sexual intercourse that cause harm, illness, and disability: having sex standing up, sex while the couple is lying on their side, sexual intercourse in the bathhouse, and the position where the woman is on top. Although the author states that it is permissible from the religious aspect for men and women to look at each other’s genitals during intercourse, he does not recommend that a man looks at his wife’s genitals during sex. He claims that the boy born due to this sexual intercourse would lack eyes. In addition, he claims that if a woman stimulates a man’s genital with her mouth and then intercourse happens, the boy to be born would be weak and weary.

Unlike the general acceptance of the period that “too much sexual intercourse is harmful,” the author states in folio-10b that there is no limit to the frequency of sexual intercourse. Then, the author specifies 6 pleasures for people: the senses of sight, hearing, taste, smell, and touch, and the sixth one is sexual intercourse. He explains that all senses and pleasures come together in sexual intercourse.

From folio-6a to 8a, the author explains the topic of sexual intercourse timing and frequency. He argues that all seasons are excellent for sexual intercourse and that there is no limit to it; however, he suggests that spring is a more suitable time for this activity. It is explained that the weather is warm in the spring, and the blood is warm and soft at work. The author states that blood is the bearer of the soul, and there will be more blood in this season. Finally, he argues that power increases with the increase of blood that provides warmth to the animals. Additionally, he holds that most people prefer to have sex at night rather than daytime, which he calls a mistake, asserting that it is better to have sexual intercourse during the day than at night except for a sense of shame.

Then the author explains which days sexual intercourse was recommended and on which days it was discouraged. Finally, it was claimed that children born due to sexual intercourse on certain days might be exposed to different diseases or be ill-natured/immoral. In contrast, children born out of intercourse on certain other days would be good, beautiful, moral, virtuous, wise, and so forth.

Folio-9b explains that if a man has sexual intercourse on a day when he has been working hard and is very tired, his heart will work beat hard (possibly referring to tachycardia). It is understood that sexual intercourse is not recommended in such periods.

### Protective, Supportive, and Therapeutic Applications for the Protection of Sexual Health

In this part, the author recommends many foods and compounds or specific drugs and ointments to enhance sexual stamina and avoid erectile dysfunction. He also tries to find solutions to other sexual health problems related to men and women.

On folio-18a, the author states that some habits are harmful to male sexual health, weaken the male genitalia, and recommends avoiding them. The list of these habits includes: Delaying urination until after intercourse despite feeling the urgency, rushing around and walking too much, sweating profusely in the bathhouse, being awake at night too long, riding horses too much, vomiting and diarrhea, having intercourse while lying on the left side or while standing up, consuming acidic foods, drinking too much hot water. He recommends that people who do not want to lose sexual potency stay away from such habits.

On folio-16b, the author recommends using pastes containing saffron, ginger, galangal, and piper longum for cases such as the reduced quantity of semen, delayed ejaculation, and weakness of the sperm. Additionally, some more foods to increase semen amount and strengthen the sexual organ are suggested on folios-18b and 19a, including carrot, chickpeas, broad beans, onion, ginger, parsnip, long pepper, fresh milk, date, mustard, and leeks.

Another recommendation to increase sexual strength and sperm production in the bahname is using foods and nuts containing animal and plant proteins. Folio-19a gives examples of these foods, including red or white meat, animal foods, such as eggs and milk, and dried fruit like pine nuts, hazelnuts, almonds, coconut, and sesame seeds. On folio-19b, various non-compound drugs/foods are listed that increase sexual potency. Of these, saffron, galangal, and cinnamon are also recommended in other parts of the bahname. In addition, other simple drugs such as amber, clove, and poppy are mentioned in the bahname.

Chapters 5 and 6 recommend oral medications and foods to increase sexual prowess and amount of semen. As can be easily understood from the bahname’s chapter index, these medications involve all kinds of applications such as solid food, oral pastes, beverages, creams, and ointments to be applied to the soles of the feet or the genital organs, suppositories, enemas, and sublingual pills to be given to the patients. Different extracts from plants, animals, and organs and various formulations of their mixtures are used for similar purposes.

Chapter 7 includes drugs and preparations in the form of ointments, creams, pomades, and plasters to be used to strengthen erection. There are many substances in these ointment preparations similar to those mentioned earlier in the bahname. Additionally, it is stated that if sexual intercourse occurs following the application of a mixture of rosewater, musk, and cinnamon to the penis as a topical, both man and woman will enjoy this relationship to the full.

Folio-27b states that topical ointments should generally be applied to the penis, groin area, and scrotum. However, chapter 10 describes topical mixtures prepared for erection strengthening for application under the soles of the feet and between the toes, an area outside the urogenital region.

On folio-27a, we see another method of drug application, in which suppositories are inserted through the urethra for erection strengthening. In chapters 12 and 13, suppositories and enemas increase sexual power, and different herbal and animal-derived preparations are described.

In chapter 8, sublingual drug use is mentioned as a particular drug application, indicating that the drug placed under the tongue can affect the target organ. In addition, the author also describes belts and ceintures made of different materials, fabrics, animal skins, or fur/wool in the fifteenth chapter for strengthening sexual potency or treating erectile dysfunction.

Chapter 16 describes drug applications to make the vagina warm and soft like a virgin girl. These treatment methods include sitting in the liquid that has been prepared from some drugs or soaking a woolen tampon in the liquid and then inserting it into the vagina.

The last section of the bahname is intended for women. The author says that particular medication absorbed by the wool and applied to the vagina in suppositories will help conceive immediately.

In chapter 11, substances and compounds that increase the pleasure of sexual intercourse are described. The author recommended applying the herbal mixtures described in this chapter by rubbing them into the penis with saliva before sexual intercourse. Finally, chapter 7 mentions the subject of priapism. The author recommends washing the erect penis with cold water as a treatment for this case.

## Discussion

It is assumed that this work was written in the thirteenth century and translated into Turkish in the fifteenth century. Although bahname was compiled about 800 years ago, we see that many of the foods and drugs or practices in bahname are still valid today. However, some of the recommendations put forward in the bahname according to the medical theories, belief values, and observational findings of the period contradict today’s scientific knowledge and data. Suggestions and preparations of drugs and food reported earlier will be discussed in the following section in comparison with the current medical literature.

First, unlike the general acceptance of the period, there is no taboo concerning the ways of sexual intercourse in bahname. It is also stated that it is religiously permissible and lawful for husband and wife to have sexual intercourse as they wish. Similar to bahname, current literature confirms that it is beneficial and sometimes necessary for the sexual happiness of husband and wife to excite and satisfy each other and to perform sexual activity in every position as they wish.^7^

Although there is no prohibition on the forms of sexual intercourse in the bahname, it is claimed that some positions are harmful for various reasons in the fourteenth chapter. There is a remarkable example: it consists of intercourse of the man lying on his back and a woman positioned on top of him. Damages claimed to occur in this position include diseases due to the discharge of female sexual secretions onto the penis and the genital area of the man, bladder pain and injury, liver swelling, and the spread of these complaints to other organs. All these justifications are factually inaccurate and cannot be confirmed by current information and data. On the contrary, the type of intercourse described in which the woman takes a riding position is recommended, especially to treat dysfunctions such as premature ejaculation.^7^ Finally, sexology and sexual psychiatry experts maintain that any position desired and enjoyed by a healthy couple can be practiced, and there is no position that is harmful to health.^8,9^

Although it is permissible from a religious perspective, the author stated that it is not considered appropriate for a man to look at his wife’s genitals during sex. It is claimed that the boy born as a result of this sexual intercourse will be lacking eyes. Additionally, it is claimed that if a woman stimulates a man’s genital with her mouth and then intercourse happens, the boy to be born will be weak and weary. We can easily say that these are nothing more than expressions of superstition and nonsense.

The author criticizes the attitude that too much sexual intercourse is harmful; moreover, he accuses those holding this view of being ignorant. Current literature leaves the spouses wholly free and does not set any limits in terms of sexual frequency. In addition, false ideas such as the suggestion that too much sexual intercourse will harm male health or that every man has the capacity for a certain limited number of ejaculations throughout his life are no longer accepted.^7^ Besides, the author states that there are 6 pleasures for people; 5 of them are the senses of sight, hearing, taste, smell, and touch, and the sixth one is sexual intercourse. He points out that all senses and flavors are coming together in sexual intercourse. From this observation, it can be concluded that all 5 sense organs should be used in intercourse, and the current literature also confirms this.

The author does not limit or prohibit sexual intercourse timing; however, the work suggests the spring and the daytime compared to other times. Current literature confirmed that the level of androgen hormones, which provide libido and increase sexual activity, rises in spring and during the day in the diurnal rhythm of the male.^10^ Additionally, current literature revealed that testosterone levels, sexual activity, and the number of ejaculations decrease in winter.^11^

The author claimed that children born due to sexual intercourse on certain days would be good-natured and healthy; by contrast, he claimed that they would be sick and ill-natured on certain other days. The interpretations in this chapter are based on the theory of the 4 senses of humor or astrological knowledge and beliefs in those times. Based on the current literature, we can say that the statements in this chapter are completely baseless superstitions.

On folio-18a, the author recommends abandoning some habits to protect male sexual health. It is claimed that certain behaviors, such as delaying urination after sexual intercourse despite feeling the need, running constantly and walking too much, sweating profusely in the bathhouse, or staying awake at night too long, when becoming repetitive practices and habits weaken the male genitalia. Another behavior that the author recommends avoiding is exaggerated horse riding. An activity today that could be compared to horse riding is the use of bicycles. Indeed, modern urology shows that prolonged cycling can adversely affect erectile function. Just like riding a horse, cycling is a chronically traumatizing factor for the perineum. In a meta-analysis investigating the relation between cycling and erectile dysfunction, Gan et al^12^ stated a positive correlation between cycling and erectile dysfunction. The argument that horse riding for long periods of time would reduce erection due to chronic trauma caused during that time can only be confirmed by careful observation and from a perspective based on experience.

The work points out that if a man has sexual intercourse on a day when he is overworked and tired, the heart will work hard (possibly referring to tachycardia). Therefore, the author does not recommend having sexual intercourse during such periods. Although this statement cannot be considered completely correct with current knowledge, modern urology has shown that there is a close relationship between sexual activity and heart rhythm and blood pressure. Sexual activity was found to be equivalent to climbing a 2-story ladder in 10 seconds in terms of exercise load and the cardiovascular system’s capacity. In this case, although the heart rate remains below 130 beats and systolic blood pressure is below 170 mmHg, the workload required during sexual activity increases in the presence of old age, obesity, cardiovascular disease, and excessive food and alcohol consumption.^13^ Similar to the literature, sexual activity was determined in the bahname as an action that strains the capacity for expending energy and the risk of being tired and developing weakness during coitus.

Many simple or compound drug formulations and foods for preserving sexual health, increasing sexual power and semen amount, and treatment of erectile dysfunction are recommended in the bahname. Vegetables such as carrots, chickpeas, broad beans, onions, ginger, parsnips, long pepper, milk, dates, mustard, and leeks; foods containing animal and vegetable protein (red or white meat varieties, eggs, milk, legumes, etc.), and dried fruits such as pine nuts, hazelnuts, peanuts, almonds, coconut, and pastes containing saffron, ginger, galangal, and long pepper to obtain such benefits are recommended in several chapters. It is possible to find studies in the current literature confirming the bahname about almost all of the foodstuffs listed in bahname. Current scientific information on a few of these will be presented here:

Carrot (*Daucus carota*), mentioned in different chapters, was proven to be positively contributing to sexual functioning by increasing the level of sex hormones in men and women and to be beneficial for all parameters of female sexual functions such as desire, arousal, orgasm, and satisfaction,^14^ to increase testosterone levels in men, trigger sperm production, and increase the reserve of sperm cells in the tail of the epididymis.^15^

It has been revealed that saffron (*Crocus sativus*) improves erection quality in men^16,17^ and also positively affects sperm morphology and motility.^18^

Ginger (*Zingiber officinale*) has an aphrodisiac effect by increasing the blood flow to the testicles, sperm count and motility, testicular volume, and serum testosterone levels.^17^ Additionally, Stein et al^19^ reported that ginger (*Zingiber officinale*) significantly improves erection quality and sexual satisfaction in middle-aged and older men.^19^

Similarly, Galangal (*Galanga officinalis*) increases the percentage of normal sperm, vitality, motility, and testosterone levels^20^; administered orally, according to the current literature, it significantly increases the total motile sperm count.^21^

Two separate experimental studies conducted with clove (*Syzygium aromaticum*) revealed that sexual activity was significantly and sustainably increased in male rats receiving clove. Furthermore, it positively affected sexual behaviors in male mice.^22,23^ Thus, certain current studies support observations found in the bahname in terms of medicaments.

The plant known as *Papaver somniferum* in Latin, sometimes called opium in the bahname and sometimes poppy, causes the smooth muscles of the corpus cavernosum to relax and triggers a strong erection due to papaverine.^24^ As a breakthrough development in modern urology and andrology, achieving an erection by injecting papaverine into the spongy tissue of the penis has been in clinical practice since the 1980s.^25^ The papaverine injection not only induces an erection but also leads to a longer than usual duration.^26^ Papaverine was also beneficial in topical application to the penis and the genital area.^27^ Moreover, papaverine injection is also featured in leading publications in urology/andrology in current reviews regarding the treatment of erectile dysfunction.^28,29^ In addition to these selected examples from the bahname, we can say as a general assessment that the positive effect of almost all of the herbal or animal extracts and preparations proposed is in line with current scientific data.

The author recommended various administration methods for the different plant, animal, and organic extracts and mixtures. Furthermore, these cures were in different forms such as solid food, oral paste, beverage, cream, and ointment to be applied to the soles of the feet or genitals, suppositories, enemas, and sublingual pills. Especially, the sublingual application is quite remarkable, considering the current scientific knowledge. In an age when the physiological absorption and action mechanisms were not yet scientifically known, proposing this method based on the absorption of drugs under the tongue to create an effect on the target organ was possible only as a result of the experiment, observation, and inference.

Chapter 7 explains the topical applications that work by a transdermal mechanism of action with ointments, creams, pomades, and plasters used to strengthen erection. Since the 1990s, positive results were obtained by applying a topical gel containing the active substance to the penis and the genital area in human patients and animal experiments. These substances include papaverine^27^ and prostaglandin E1.^30^ Current literature found that topical application of both agents increases the penile blood flow significantly, and potent erections are achieved after topical application. Various reviews indicate that oral therapy is a promising method for groups of patients who cannot be treated by intra-cavernous injection due to drug interactions or non-responsiveness, needle fear.^31,32^

Another remarkable drug administration method recommended to support erection is the application of a preparation in the form of suppositories to be inserted through the urethra. Since the 1990s, preparations containing prostaglandin E1 have been administered through the urinary tract with this method, which is seen as an ideal and practical treatment of erectile dysfunction today, known under the name of MUSE.^33,34^ Although its effectiveness is not as great as intra-cavernous application, its ease of use and non-invasiveness make this a preferred method.^35^ In another study, Padma-Nathan et al. recommended a topical cream containing prostaglandin E1 to be dripped into the urethral orifice. .^33^ Similarly, topical gels are applied to the penis, perineum, and testicles in modern transdermal erection therapy.^27^

Chapters 16 and 17 include a recipe intended to make the vagina warm, soft, and even as tight as that of a virgin girl, administration of the liquid prepared from some drugs was suggested either in the form of a bath to sit in or by inserting a woolen tampon soaked in the liquid into the vagina. Current medical knowledge and scientific literature do not include any pharmaceutical or herbal drugs having such an effect.

Similarly, the seventeenth chapter says that some drugs soaked up by wool and then inserted into the vagina will ensure pregnancy immediately. Such a practice is not found in modern science. In addition, the author claimed that if a man applies tar or sesame oil on his penis before sexual intercourse, the woman will not conceive, or even if she becomes pregnant, she will have a miscarriage. There is no corresponding information in the current literature.

The bahname also described the artificial penis used by women to masturbate, which is known as zıbık in the Middle East and dildo in Western languages. Similarly, modern sexology suggests the dildo using for therapeutic purposes. Another version of a dildo that increases pleasure through vibration is a vibrator subjecting many scientific studies. Herbenick et al^36, 37^ reported that sex devices such as vibrators and dildos are frequently recommended to patients; another article stated that vibrators are an important option in the hands of clinicians to increase sexual function and respond to certain sexual problems. Among the therapies used in treating sexual dysfunction, especially female orgasm and arousal disorder, it is reported in today’s scientific literature that erotic devices, including dildos and similar items, contribute positively to the solution of these issues.^38^

In case the erect penis did not soften again, the author recommended washing with cold water. Similar to bahname, the first step in priapism treatment is the application of cold compresses, and in some cases, this simple intervention can achieve the desired softening.^39,40^ Pryor et al^41^ also suggest cold compresses or a cold shower as the first aid method that can be applied by the patient himself or by auxiliary health personnel. Interestingly, a similar method was proposed in the bahname six centuries ago.

The limitations of our study were as follows: first, we analyzed an old original manuscript in our study, so some words were erased over time and could not be read. However, these parts are insignificant in the whole text and do not affect the semantic integrity of the work. Second, some plant namings made in the historical period when the work was written are not used today and cannot be found in the current literature.

In conclusion, we investigated the fifteenth-century translation by Musa b. Mes’ud of the *Bahname-i Padişahi*, which is the oldest known Turkish example of a bahname. We discussed the preventive, supportive, and therapeutic practices associated with sexuality, sexual life, and sexual health issues mentioned in the bahname and analyzed the suggested treatments and recommendations from a period of about 7 centuries in the light of the current literature.

The author synthesized empirical information with experiences from the past in the bahname while dealing with sexual life and sexual health issues. These issues are generally evaluated in the context of health; a religious perspective is also provided when needed. In particular, the author’s recommendations on sexual health and herbal or animal drugs are consistent with the current literature. The work must have been written as a result of careful observation and profound experience. In addition, we can say that the author went beyond the generally accepted beliefs he lived in, especially on issues of sexual life. Nevertheless, some of the information and suggestions included in the book are entirely irrational and unscientific in the light of the current literature.

Finally, this study is an original and novel investigation of the bahnames, which have not found the attention they deserve in today’s academic studies, although they are an essential part of the Turkish-Islamic culture. We revealed all critical information in the bahname and compared it with Turkish and international sources in the current literature on urology, pharmacology, andrology, and sexology. There is no other study examining the bahnames with this method. Thus, we believe that our work will be a significant contribution to the research literature.

This manuscript is an English translation of the article titled “Osmanlı Tıp Literatüründe Türkçe Yazılan İlk Bahnâmenin Güncel Üroloji Açısından Değerlendirilmesi (in Turkish)” published previously in the Anatolian Clinic the Journal of Medical Sciences with DOI number “10.21673/anadoluklin.1076048” in May 2022, to reach new readers in a different language. The copyright and publishing consent was approved by the Anatolian Clinic the Journal of Medical Sciences (no. HSV-AK-2022-001).

## Figures and Tables

**Table 1.** The Index of Bahname Quoted by Uzel with Reference to Sehsuvaroglu

Chapters	Title
Chapter 1	Body Temperaments and Their Symptoms
Chapter 2	Foods Strengthening Sexual Intercourse
Chapter 3	Simple Foods
Chapter 4	Compound Foods
Chapter 5	Drinks
Chapter 6	Pastes
Chapter 7	Ointments
Chapter 8	Pills
Chapter 9	Girdles and Belts
Chapter 10	Drugs Applied to the Soles of the Feet
Chapter 11	Drugs Enhancing Sexual Appetite
Chapter 12	Enemas for Strengthening Sexual Intercourse
Chapter 13	Powders
Chapter 14	Sex Positions
Chapter 15	Penis-Enlarging Drugs
Chapter 16	Drugs Making the Vulva Tight and Soft
Chapter 17	Contraceptive Drugs
